# Assessment of Exercise Capacity and Oxygen Consumption Using a 6 min Stepper Test in Older Adults

**DOI:** 10.3389/fphys.2017.00408

**Published:** 2017-06-14

**Authors:** Siana Jones, Therese Tillin, Suzanne Williams, Emma Coady, Nishi Chaturvedi, Alun D. Hughes

**Affiliations:** Cohort Phenotyping, Institute for Cardiovascular Science, University College LondonLondon, United Kingdom

**Keywords:** exercise, stepper test, oxygen consumption, aging

## Abstract

It is often necessary to assess physical function in older adults to monitor disease progression, rehabilitation or decline in function with age. However, increasing frailty and poor balance that accompany aging are common barriers to exercise testing protocols. We investigated whether a 6-min stepper test (6MST) was acceptable to older adults and provided a measure of exercise capacity and a predicted value for peak aerobic capacity (VO_2max_). 635 older adults from a tri-ethnic UK population-based cohort were screened to undertake a self-paced 6MST. Expired gas analysis, heart rate and blood pressure monitoring were carried out. A sub-set of 20 participants performed a second 6MST for assessment of reproducibility and a further sub-set of 10 performed the 6-min walk test as verification against a well-recognized and accepted self-paced exercise test. 518 (82%) participants met inclusion criteria and undertook the 6MST (299 men, mean age 71.2 ± 6.4). Step rate showed a strong positive correlation with measured VO_2_ (*r* = 0.75, *p* < 0.001) and VO_2_ was lower in women (male-female difference in VO_2_ = 2.61 (95% confidence interval −3.6, −1.7) ml/min/kg; *p* < 0.001). 20 participants repeated a 6MST, step rate was higher in the second test but the predicted VO_2max_ showed good agreement (mean difference = 0.1 [3.72, 3.95] ml/min/kg). In 10 participants who completed a 6MST and a 6-min walk test there was a strong positive correlation between walking rate and step rate (*r* = 0.77; *p* < 0.009) and weaker positive correlations between the tests for measured VO_2_ and peak heart rate. In conclusion, the 6MST is a convenient, acceptable method of assessing exercise capacity in older adults that allows VO_2max_ to be predicted reproducibly. The test shows good correlation between performance and measured physiological markers of performance and can detect the expected gender differences in measured VO_2_. Furthermore, the 6MST results correlate with a previously verified and established self-paced exercise test.

## Introduction

Objective methods for assessing exercise capacity are an integral component for monitoring changes in physical function which are known to decline with age and disease.

The gold standard for assessing exercise capacity and aerobic fitness involves an exhaustive cardio-pulmonary exercise test (CPET) to provide a measure of oxygen consumption at peak exercise (VO_2max_). VO_2max_ is the gold standard marker of cardio-respiratory health which can predict cardiovascular morbidity, cardiovascular mortality and all-cause mortality (Kokkinos et al., 2009; Reddigan et al., 2012). However, exhaustive CPET may not be acceptable to older individuals, in whom up to 50% may be unable or unwilling to undertake maximal exercise testing (Wetterqvist et al., 2002). Moreover, in older adults achievement on these testing protocols may not represent real-life functionality (Greig et al., 1993; Huggett et al., 2005) and exercise to exhaustion is often not achieved (Church et al., 2008). Exhaustive CPET are also time-consuming, require specialist exercise laboratory testing facilities and, as they carry risk of adverse events, necessitate trained medical supervision. Submaximal exercise testing is a valuable alternative (Noonan and Dean, 2000) and numerous ways of predicting maximal oxygen consumption (VO_2max_) from submaximal test results have been described (Smith et al., 2016).

In older adults barriers to performing well on exercise testing protocols are often related to poor balance and frailty (Parvataneni et al., 2009). Self-paced walking tests have previously been well tolerated by older adults in population studies (Simonsick et al., 2001; Lange-Maia et al., 2015) but require a long corridor (ATSstatement, 2002) and are not conducive to accurately measuring physiological changes during the exercise period, for example, blood pressure.

A 6-min stepper test (6MST), conducted on an upright stepper ergometer, was previously developed for patients with chronic obstructive pulmonary disease (COPD; Dal Corso et al., 2007). There are several benefits of this test: first, the stepper test does not require a corridor or similar space which is often limited in clinical and research settings. Second, a stepper allows a wider range of physiological responses to be measured during exercise. For example, accurate assessment of exercise induced blood pressure changes can be carried out during a stepper test using devices that cannot be used reliably during walking. And third, a stepper test can be used to assess exercise capacity in frail elderly subjects or those with poor balance by using support rails.

Borel et al. concluded that the stepper test was reproducible and well tolerated in COPD patients and distinguished between healthy individuals and patients with COPD (Borel et al., 2010). The test has since been shown to correlate with the 6-min walk test (6MWT) and maximal exertion CPET in a larger study of COPD patients (Coquart et al., 2015; Grosbois et al., 2016).

The feasibility of this test for a population-based sample of older adults, with no upper limit for age, has never previously been assessed. Furthermore, although free-standing step tests have been advocated for the prediction of maximal aerobic capacity (Bennett et al., 2016), the stepper test has not previously been used for this purpose.

The objectives of this study were to; (1) investigate the 6MST as a test to assess exercise capacity in an older population of adults (2) evaluate intra-test agreement and compare with an established self-paced exercise test, the 6-min walk test (6MWT), and (3) utilize established equations to predict VO_2max_ from the sub-maximal heart rate measured during the test.

## Methods

### Participants

Participants in this study were recruited from the Southall And Brent REvisited study (SABRE). SABRE is a population-based longitudinal tri-ethnic cohort study of individuals who were resident in West London, UK; participants were originally recruited in 1988 (Tillin et al., 2012) Participants and spouses or partners of participants, attending the 3rd SABRE follow-up clinic visit, were invited to undertake the exercise test unless exclusion criteria were met. Exclusion criteria were based on the ATS/ACCP guidelines (American Thoracic and American College of Chest, 2003) and included; angina or a recent cardiovascular event (MI, stroke, TIA), an uncontrolled arrhythmia, uncontrolled arterial hypertension, severe aortic stenosis, severe symptoms of COPD and any orthopedic impairment severely compromising exercise performance.

All procedures were in accordance with the principles of the Helsinki declaration, all participants gave written informed consent and the study was approved by the National Research Ethics Service (NRES) Committee London—North Fulham.

### Exercise protocol

**Figure 1:**
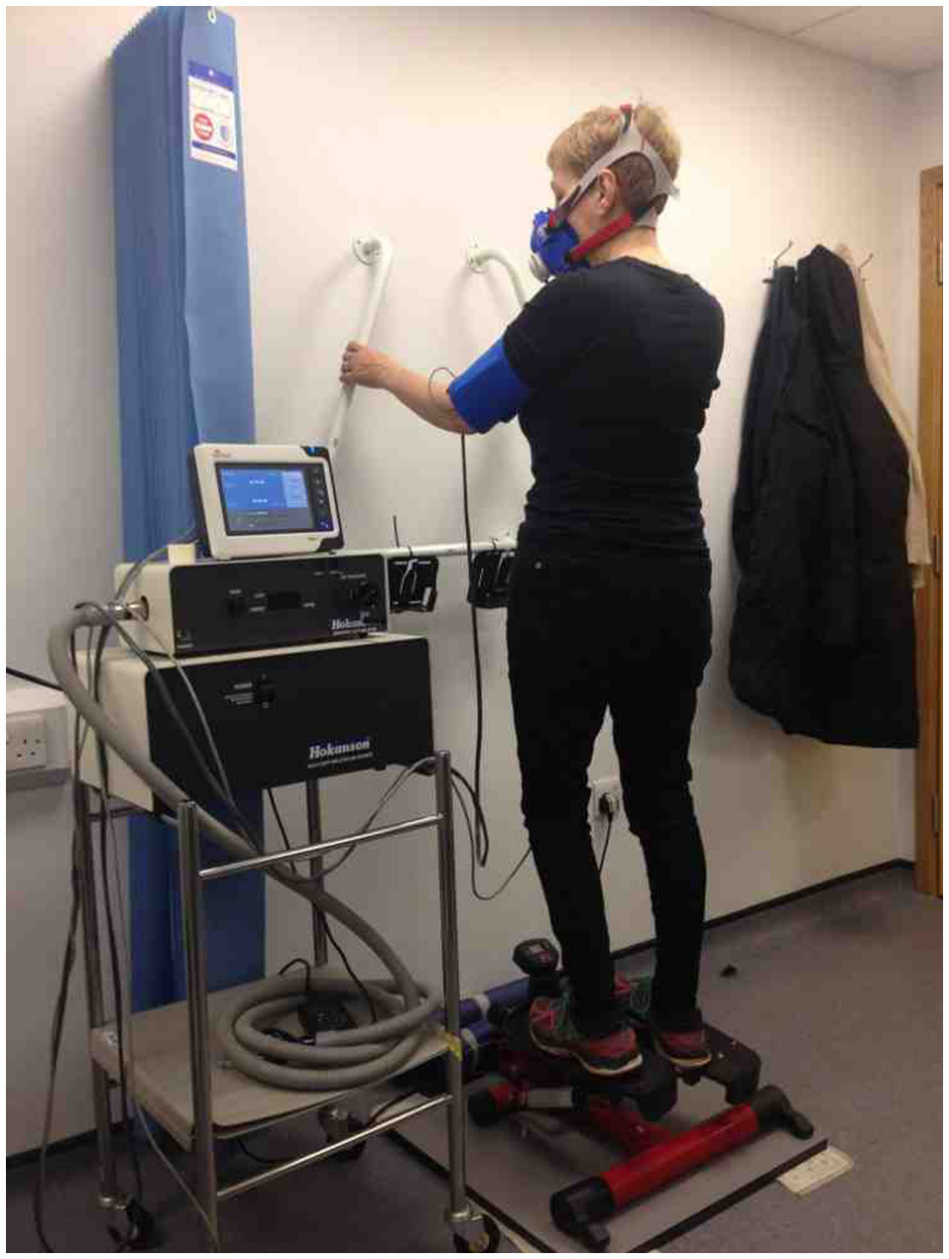
Example set-up for the 6-min stepper test including blood pressure monitor and gas analysis mask.

The exercise test was a self-paced, 6MST (Homcom, miniStepper; Figure 1). The testing protocol was described to participants using the following instructions:

- The objective of this test is to complete as many steps as possible within 6 min
- Start at a pace you feel you could continue at for 6 min and try to maintain this pace
- If you become exhausted or experience dizziness or chest-pain please stop immediately

Prior to undertaking the test participants were permitted to familiarize themselves with the stepping procedure, all participants were permitted to use the custom-built wall support for balance during the test if necessary. Termination of the test prior to 6 min was made based on ATS/ACCP guidelines (American Thoracic and American College of Chest, 2003) or because the participant reported intolerable dyspnea or muscle fatigue. Duration was not limited by heart rate in line with previous research (Jain et al., 2011). At the end of the test, participants were asked to grade their perceived level of exertion on a modified (0–10) Borg scale where 0 is resting and 10 is maximal exertion(Kendrick et al., 2000). The number of steps completed and duration was recorded.

### Physiological measurements

Heart rate and expired gas variables were measured using a heart rate monitor and portable gas analyser (K4b^2^, COSMED). Measured oxygen consumption (VO_2_, ml/min/kg) was calculated as the highest value determined from a rolling 60-s average calculated across the duration of exercise.

Peak heart rate was the highest measured heart rate during the exercise phase. Predicted maximal heart rate (maxHR) was estimated using two Equations (1) and (2) commonly used in the literature (Fox and Naughton, 1972; Tanaka et al., 2001):

(1) $$\begin{array}{c} maxHR(Fox)=220-age\;(yr) \end{array}$$

(2) $$\begin{array}{c} maxHR(Tanaka)=208-0.7\times age\;(yr) \end{array}$$

Blood pressure was measured using a motion-insensitive validated device (Tango M2, SunTech) at rest, 2, 4, and 6 min following the start of exercise, immediately post-exercise termination and at 3 min recovery.

### Predicting maximal oxygen consumption

Maximal oxygen consumption (VO_2max_) was estimated using predictive equations for men, equation 4 (von Dôbeln et al., 1967). This equation was adjusted for women based on previously collected data (Astrand, 1960) (Equation 4):

(3) $$\left(Men\right)VO_{2}max=1.29\cdot \sqrt{\frac{Load\;[kg\;.\;m\;.\;min^{-1}]}{Peak\;heart\;rate\;[bpm]-60}}\cdot e^{-0.0088}$$

(4) $$\left(Women\right)VO_{2}max=1.18\cdot \sqrt{\frac{Load\;[kg\;.\;m\;.\;min^{-1}]}{Peak\;heart\;rate\;[bpm]-60}}\cdot e^{-0.0090}$$

The workload was calculated from stepping rate (steps/min), step height (meters), which was constant at 15 cm for all tests, and participant weight (Kg), Equation (5).

(5) $$\begin{array}{l} Load(Kg.m)=(step\;rate\;\cdot step\;height\cdot Wt+5)\\ \;+\;(\frac{step\;rate\cdot step\;height\cdot Wt\;+\;5}{3}) \end{array}$$

We excluded participants from this analysis who were medicated with a β-blocker (*n* = 79) or who did not achieve a heart rate of at least 95 bpm during exercise (*n* = 25). The percentage of predicted VO_2max_ during the exercise was calculated using the highest VO_2_ measured during the test.

### Test-re-test reproducibility

A sub-set of 20 participants completed a second 6MST within 1 week of the first test, including physiological measurements, to assess test-re-test reproducibility. Number of steps completed, perceived exertion, heart rate, systolic blood pressure in the final minute of exercise, measured VO_2_ and predicted VO_2max_ were compared between the first and second 6MST.

### Six minute walk test (6MWT)

A further sub-set of 10 participants also completed a standard 6MWT, as described (ATSstatement, 2002). In brief, participants were directed to walk up and down a long corridor at a pace as fast as could be maintained without running. Their objective was to walk as many lengths of the corridor a possible.

Number of steps or distance walked, perceived exertion, measured heart rate and highest measured VO_2_ were compared between the 6MST and the 6MWT.

### Statistical analysis

Categorical data are presented as n (%). Continuous data were examined for normality and participant characteristics are presented as means ± standard deviation or median (interquartile range) if skewed; other results are presented as means (95% confidence interval) after log transformation of skewed data. Reproducibility data were assessed using Bland-Altman plots and are presented as mean differences [limits of agreement (LOA), i.e., ± 1.96 × standard deviation of differences]. Correlations were assessed using Pearson's correlation coefficient. Student's *t*-tests or analysis of covariance was used for statistical comparisons. Statistical significance was assigned if *p* < 0.05.

## Results

### Participants

Six hundred and thirty five participants underwent screening for exercise testing, of these 518 (82%) undertook the 6MST. Expired gas analysis was possible in 476 (92%) participants who performed the exercise test. Gas analysis was not possible in 8% of participants because of a technical problem with the equipment or because the participant declined to wear the face mask. Participant characteristics and exercise performance, stratified by gender, are shown in Table 1.

**Table 1:** Characteristics, exercise performance and physiological response (mean ± SD) for 518 participants who undertook the 6-min stepper test and the 476 who had expired gasses analyzed.

**Characteristic**	**Total (518)**	**Men (299)**	**Women (219)**	***P*-value**
Age (years)	71.2 ± 6.4	73 ± 5.4	68.8 ± 6.8	<0.001
Height (cm)	165.6 ± 8.9	170.7 ± 7	158.6 ± 6.2	<0.001
Weight (Kg)	75.8 ± 12.8	79 ± 12	71.1 ± 12.6	<0.001
Steps completed	197 ± 78	213 ± 77	175 ± 72	<0.001
Steps/minute	38 ± 10	40 ± 10	35 ± 10	<0.001
Peak Borg	5.3 ± 2.2	5.1 ± 2.2	5.5 ± 2.1	0.083
	(*n* = 476)	(*n* = 278)	(*n* = 198)	
Peak VO_2_ (ml/min/kg)	15.8 ± 4.1	16.89 ± 4.1	14.33 ± 3.59	<0.001
Peak HR (bpm)	123 ± 24	120 ± 23	127 ± 25	<0.001
Percent maxHR achieved (%)	83 ± 16	82 ± 16	84 ± 16	0.064

*The p-value refers to the unadjusted comparison of men and women using a t-test*.

One hundred and seventeen participants did not undertake the exercise test. Only 4 participants declined to exercise. The remaining 113 participants were excluded for the following reasons: uncontrolled hypertension (*n* = 40), severe arthritis (*n* = 17), non-arthritic mobility-related limitations (*n* = 20), recent episode of angina or cardiovascular event including TIA, stroke or MI in the past 6 weeks (*n* = 18), uncontrolled arrhythmia (*n* = 10), no trained staff present to conduct the test or lack of time during the visit (*n* = 5), severe COPD (*n* = 1), recent aortic root repair (*n* = 1) and visual impairment (*n* = 1).

Three hundred and sixty three participants (70%) completed 6 min of stepping. Reasons for terminating the test before 6 min were: excessive systolic or diastolic blood pressure (>230 or >115 mmHg, respectively; *n* = 43), a sudden blood pressure drop (*n* = 1), instability (*n* = 3), excessive dyspnea (*n* = 18), muscle fatigue (*n* = 29), general fatigue or arthritic joint/back pain (*n* = 61).

**Figure 2:**
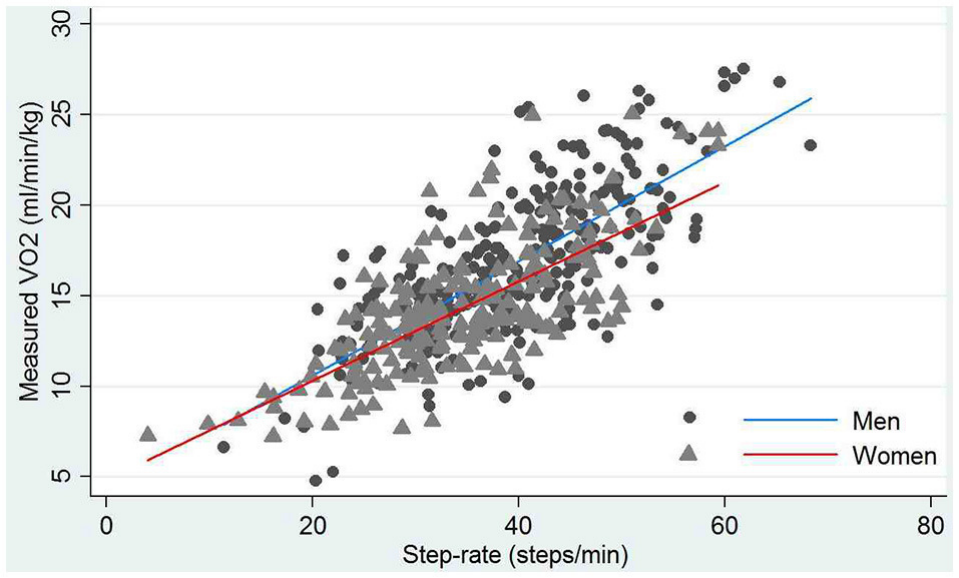
Correlation between step rate and the highest measured oxygen consumption (VO_2_) during exercise stratified by gender. Circles and triangles are individuals' data for men and women, respectively. Blue and red lines are lines of best fit for men and women, respectively.

Stepping rate and measured VO_2_ showed a strong positive correlation (*r* = 0.75; *p* < 0.001; Figure 2). Men outperformed women in terms of steps completed and measured VO_2_ (Table 1). This sex difference in measured VO_2_ remained after adjustment for height, weight and age (2.61 (95%CI −3.6, −1.7) ml/min/kg lower in women; *P* < 0.001).

### The reproducibility of the 6MST

**Figure 3:**
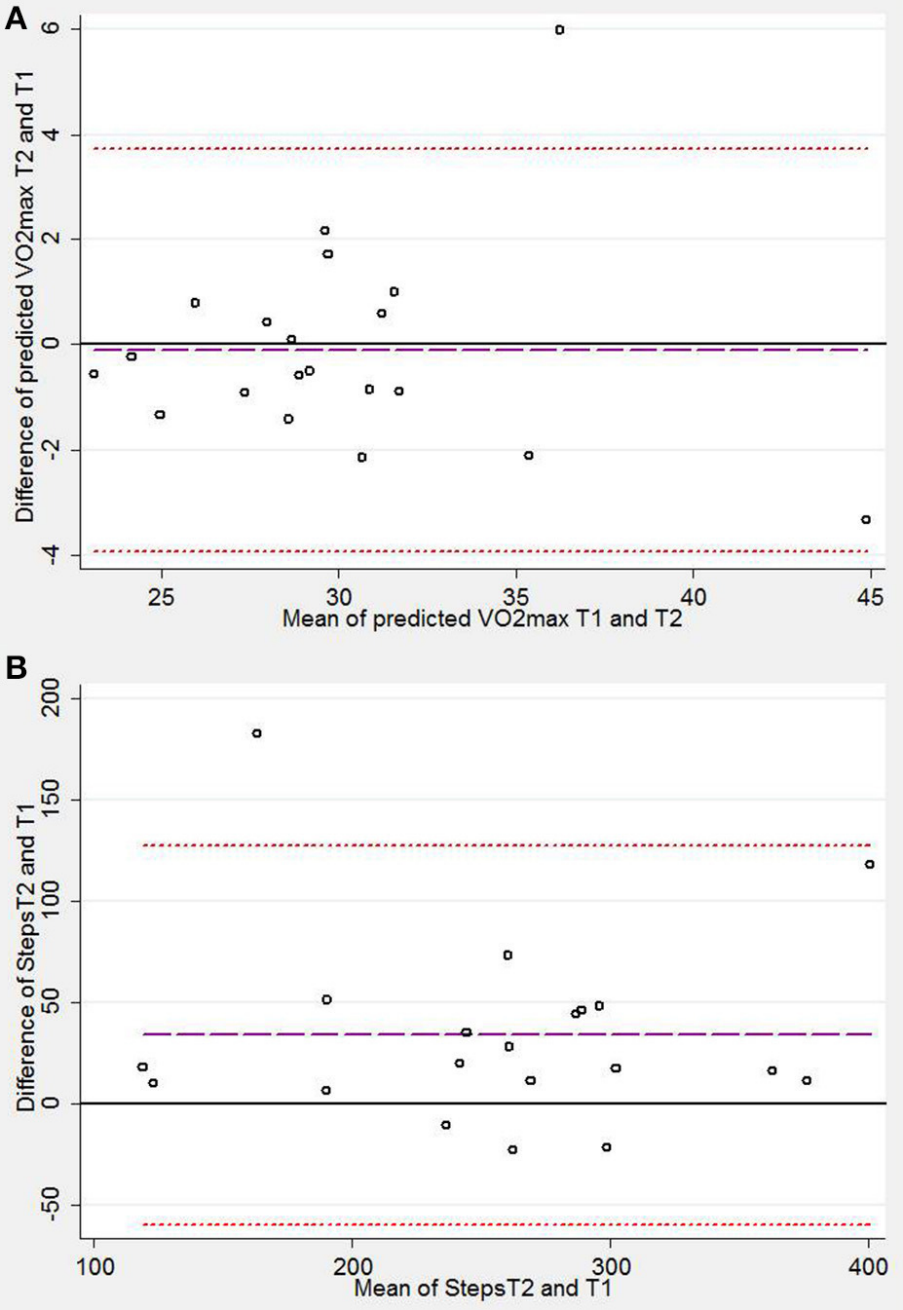
Bland-Altman plots demonstrating levels of agreement between stepper test 1 (T1) and stepper test 2 (T2) for **(A)** predicted maximum oxygen consumption (VO_2_max) and **(B)** steps completed. Mean difference is plotted as a purple dashed line, upper and lower limits of agreement are plotted as red-dashed lines. Mean difference [limits of agreement] were **(A)** 0.11 [−3.95, 3.72] ml/min/kg and **(B)**, 34[−59.8, 127.7] steps.

Twenty participants completed a repeat 6MST. On average participants completed 34 steps more in the second test compared to the first (Table 2). Figure 3B shows a Bland-Altman plot of agreement between the number of steps completed in the first and second step tests (mean difference = 34 steps, [LOA −62, 130]).

**Table 2:** Performance and physiological measures and predictions from stepper test 1 and stepper test 2.

	**Stepper test 1**	**Stepper test 2**	***p*-value**
Steps completed	242 ± 80	276 ± 79	0.005
Step rate (steps/min)	43 ± 10	47 ± 12	0.001
Perceived exertion (Borg)	6.2 ± 2.2	6.9 ± 1.8	0.03
Systolic blood pressure (mmHg)	183 ± 7	183 ± 9	0.99
Diastolic blood pressure (mmHg)	74 ± 3	75 ± 3	0.91
Measured peak heart rate (bpm)	124 ± 19	130 ± 20	0.02
Measured VO_2_ (ml/min/kg)	16.9 ± 4.9	18.1 ± 4.6	0.07
Predicted VO2max (ml/min/kg)	30.1 ± 5.0	30.0 ± 4.9	0.80

*Data are means ± SD of 20 observations; p-values calculated using a paired t-test*.

Perceived exertion, measured VO_2_ and measured peak heart rate were all higher on the second test compared to the first although the highest measured VO_2_ did not achieve statistical significance (Table 2). The mean predicted VO_2max_ did not differ between the tests and values show good agreement between the first and second stepper tests (Table 2, Figure 3A). Blood pressure measurements were possible during the final 3 min of exercise in all participants; the average systolic blood pressure in the final 3 min of exercise was not significantly different between stepper tests.

### The 6MST shows good correlation with a 6-min walk test (6MWT)

Ten participants completed a 6MST and a 6MWT. Walking rate and step rate were positively correlated (*r* = 0.77; *p* < 0.001) as were walking distance and total number of steps (*r* = 0.61). Average values for peak heart rate, measured VO2 and perceived exertion (modified Borg) were similar and measured VO2 (ml/min/kg), peak heart rate and measured VO_2_ also showed positive correlations between the 6MST and the 6MWT; however, there was no relationship between perceived exertion on the two tests (Table 3).

**Table 3:** Results of the stepper test and walk test and Pearson's correlation coefficient (r) for the two tests.

	**Stepper test**	**Walk test**	***r***	***p*-value**
Steps completed/meters walked	211 ± 89	518 ± 105	0.61	0.06
Step rate (steps/min)/walk rate(m/min)	40 ± 10	86 ± 18	0.77	0.009
Perceived exertion (Borg)	6.7 ± 1.7	5.8 ± 1.4	−0.14	0.7
Measured peak heart rate (bpm)	116 ± 21	114 ± 21	0.50	0.1
Measured VO2 (ml/min/kg)	16.5 ± 6.4	17.8 ± 5.2	0.43	0.2

*Data are means ± SD of 10 observations*.

### Predicting VO_2max_

**Figure 4:**
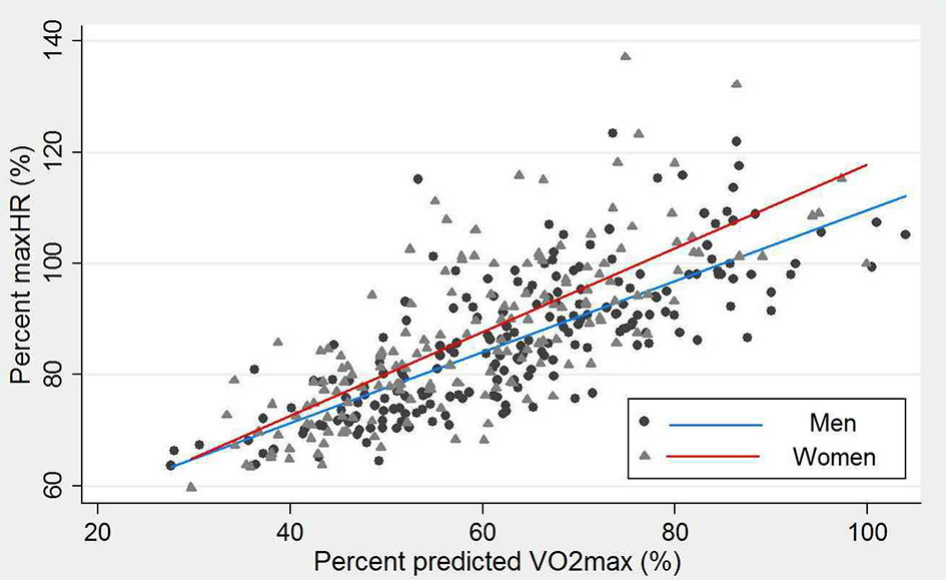
Relationship between the percent predicted maximum heart rate using (Equation 1) [maxHR(Fox)] and percent predicted oxygen consumption (VO_2_max) for men (circles) and women (triangles).

VO_2max_ was predicted for 372 participants (mean ± SD: 27 ± 4.9 ml/min/kg). Percent of predicted VO_2max_ reached during exercise was 61 ± 15%. Percent maxHR(Fox) or maxHR(Tanaka) and percent predicted VO_2max_ showed strong positive correlations (*r* = 0.74; *p* < 0.001 and *r* = 0.74; *p* < 0.001, respectively). Figure 4 illustrates this relationship for men and women separately using maxHR(Fox), results for maxHR(Tanaka) were similar.

## Discussion

This study demonstrates that the 6MST is an acceptable, valid and reproducible method for assessing exercise capacity in a population-based sample of adults over the age of 65. The test also provides an adequate sub-maximal heart rate allowing aerobic capacity (VO_2max_) to be predicted, a valuable marker of physical function that is difficult to measure directly in this population (Huggett et al., 2005).

Free standing step tests are widely used to assess exercise capacity and predict VO_2max_ (Bennett et al., 2016) but stepper tests are a less commonly described method of exercise testing. Previous studies verifying the 6MST were carried out in COPD patient populations with a younger mean age than the sample in this study (Borel et al., 2010; Grosbois et al., 2016). In this study of an older adult population who were expected to experience increased frailty and poorer balance, we fastened support rails to the wall in front of the stepper. Acceptability of the test was good, only 4 participants out of 635 declined to exercise. This compares favorably with the acceptability of maximal exercise testing using a bicycle ergometer in similar age group (Wetterqvist et al., 2002) Exclusion of the remaining 113 participants was for health related issues or safety requirements (American Thoracic and American College of Chest, 2003).

There was a strong positive relationship between performance (step rate) and measured VO_2_ suggesting the 6MST is sensitive to increases in load (Figure 2). The 6MST detected expected gender differences in performance (Wheatley et al., 2014) providing some evidence of validity.

Submaximal exercise testing with a prediction of VO_2max_ is frequently described (Bennett et al., 2016; Smith et al., 2016). In this study we used an equation to predict VO_2max_ from the sub-maximal heart rate, first described by von Dôbeln et al. (1967). We selected this equation because it included individuals up to 70 years old; unlike some alternative equations (Marley and Linnerud, 1976) Numerous equations have been reported to predict maximum heart rate with age, we chose two that have been widely used (Fox and Naughton, 1972; Tanaka et al., 2001); while all such equations have limitations, (Robergs and Landwehr, 2002) in this study they were only used to give an indication of the intensity of the sub-maximal exercise achieved using the 6MST.

### Reproducibility of the 6MST and predicted VO_2max_

Steps completed during the second stepper test improved by an average of ~14% with associated improvements in physiological markers of exertion: heart rate and measured VO_2_ (Table 2). This difference is likely attributable to a “learning effect.” Other studies report similar findings using other field tests (Uszko-Lencer et al., 2017) Despite this, predicted VO_2max_ showed very good agreement between the first and the second test (Figure 3A), suggesting that the estimate is valid during a primary test.

### Agreement of the 6MST with the 6MWT

Performance on the 6MST is measured as steps completed or step-rate. Both markers of stepping performance correlated positively with markers of walking performance; meters traveled and walking rate (m/min; Pearson's *r* = 0.61 and 0.77, respectively). Physiological measurements, VO_2_ and heart rate, were also positively correlated between the tests although less strongly. Perceived exertion, assessed as a Borg score (0–10), was scored by the participant at the end of each test. Scores after stepping were not correlated with scores after walking. One explanation may be that individuals perceived the severity of the two tasks within a limited range which reduces the correlation (Bland and Altman, 2011). It is also possible that participants experienced these different types of exercise as being more, or less, difficult for reasons unrelated to the intensity of the exercise. Further investigation into the perception of difficulty of an exercise is beyond the scope of this study but further investigation into its contribution to the acceptability of the stepping test would be interesting.

## Limitations

### Pace maintenance

The 6MST is a self-paced test with an instruction to maintain stepping pace throughout. While this allows the protocol to be simple and increases acceptability, it is not certain that the participant has maintained pace throughout the test and, therefore, kept the work load constant.

### Support rails

We fixed support rails to the wall in front of the stepper. This may also compromise the accuracy of calculating workload because we cannot be sure to what extent each participant used the rails to assist themselves during exercise. We found that all participants opted to support themselves by holding on to the rails during the test.

### Direct verification with measured PeakVO_2_

We did not directly validate the predicted VO_2max_ by conducting maximal exercise tests in our study sample. As discussed above many older people are unwilling to undertake maximal exercise testing (Wetterqvist et al., 2002) and, in those who do, exercise to exhaustion is often not achieved (Church et al., 2008). Previously, performance on the 6MST has been shown to correlate well with VO_2max_ in younger COPD patients (Grosbois et al., 2016).

## Conclusion

In conclusion, the 6MST is an appropriate method of assessing fitness that is well accepted by older adults and has some advantages compared with the 6MWT. VO_2max_ can be estimated reproducibly from the submaximal heart rate and results from the test correlate with the 6-min walk test.

## Author contributions

All authors contributed to study design, data interpretation, and revision of the manuscript. All authors approved the final version and agree to be accountable for the work, taking responsibility for the integrity of the data and the accuracy of the data analysis.

### Conflict of interest statement

The authors declare that the research was conducted in the absence of any commercial or financial relationships that could be construed as a potential conflict of interest.
